# Succinimid of Mercury

**Published:** 1916-02

**Authors:** B. L. Wright


### APPLICATION AND DOSAGE OF SUCCINIMID OF MERCURY.

BY B. L. WRIGHT, M. D.

In pyorrhoea, in the male patient, a deeper muscular injection of mercuric succinimid gr. 1 (65 mgm.) should be administered every seventh day, until the discharge of pus has entirely disappeared, and the gums have regained their normal condition and appearance. If the pockets have not been entirely obliterated and the loose teeth have not become firmly fixed by this time, they will quickly do so without further treatment, providing the hygiene of the mouth and teeth is properly carried out.

Of course, when a tooth socket has become destroyed by alveolar absorption, it is impossible for the tooth to become fixed, and it should be removed.

In female patients the dose should be from gr. 1-5 (13 mgm.) to gr. 2-5 (26 mgm.) less than that administered to males. Mercurialism of any marked degree should be met with smaller doses at succeeding injections, or, if the symptoms are severe, the mercury is discontinued until they have disappeared.

In treating cases complicated by a secondary systemic infection, the dosage and interval between injections will materially differ from the above, according to the nature of the secondary infection, whether acute or chronic, severe or mild, etc. As this question more properly belongs to the realm of internal medicine, I refer those interested to my former publications.*

I have seen several cases of pyorrhea recover completely under the above treatment without local surgical intervention, but these were those in which calcareous deposits and tartar had not formed, nor was the omission of local treatment desired by me, but due to the absence on leave of my dental colleague.

### LOCAL TREATMENT.

The local treatment as administered by Dr. P. G. White embraces the following steps: Careful expression of the pus from the pockets, thorough removal of calcareous deposits and tartar wherever found; extraction of hopeless teeth and roots, polishing of the tooth structure, followed by applying to the margin of the gums equal parts of tincture of iodin, tincture of aconite, and chloroform, continued every other day. This latter procedure may be, and has been omitted in many of our cases without any appreciable difference in the time required to effect

* "The Treatment of Diseases of Vegetable Parasitic Origin by Deep Muscular Injections of Mercury," by B. L. Wright, Medical Record, July 11, 1914.

a cure, but we consider it a valuable adjunct to the local curettage and systemic treatment.

Including the twenty-eight cases reported cured in the *Medical Record* of March 13, 1915,† we have now, on June 3, 1915, treated sixty consecutive cases, with 100 per cent. of cures and not a single recurrence.

No treatment advocated for this disease has given results which can compare with these, yet more and possibly greater things can be claimed for it, for among these sixty cases were a number which presented systemic infection in all probability secondary to the pyorrhea, viz., four cases of acute infectious arthritis—so-called acute rheumatic fever—nineteen cases of chronic infectious arthritis, one of chronic gastritis, one of chronic bilateral facial neuralgia, one of chronic laryngitis, one of chronic otitis media, and two of severe chronic lumbar myositis—lumbago—or 48.33 per cent. of the total number of cases treated, all of which were completely cured in remarkably short periods of time. In addition two had gonorrheal arthritis, which was also cured. Another had syphilitic arthritis—Wassermann 4 plus; in this case the pyorrhea was cured by four injections in sixteen days, but the syphilitic arthritis was decidedly worse—Wassermann 3 plus. The patient was then transferred to the hospital, where, following the first injection of 900 mgm. neosalvarsan, the arthritis disappeared, and the Wassermann became negative.

The high percentage—38 per cent.—of arthritic cases in this series is due to the fact that these men first reported for treatment of so-called rheumatism, and the pyorrhea was discovered when search was being made for a local focus of infection.

† "The Treatment of Pyorrhea Alveolaris and Its Secondary Systemic Infections by Deep Muscular Injections of Mercury," by Dr. B. L. Wright and Dr. P. G. White.

### NUMBER OF INJECTIONS.

The longest period of time required to effect a cure was forty-one days, the shortest, four days; average seventeen days. The greatest number of injections required to cure the primary infection in any one case was seven; the smallest number, one; average, 2.9. The diagnosis in each case was based upon classical symptoms and conditions; pus was present in every case, the presence of slight pathological changes in the gum tissue, or of any particular organism not being considered as diagnostic.

By *cure* I mean the total disappearance of pus, the gums becoming normal pink, hard, and firm, the filling-in and obliteration of pockets, and the firm fixation of loose teeth provided the alveolar processes had not been absorbed.

### CORROBORATIVE EVIDENCE.

Further proof of the value of this method of treatment is that A. A. Dent. Surgeon G. H. Reed, U. S. navy, reports treating at the navy yard, Boston, Mass., twenty-nine consecutive cases, twenty-eight of which were completely cured, while the twenty-ninth was markedly improved, when the ship to which he was attached sailed, preventing further treatment and cure.

Samuel T. Ladd, M.D., of Portsmouth, N. H., co-operating with E. C. Blaisdell, D.M.D., of Portsmouth, and several others of the same place, reports eight consecutive cases treated and cured, making in all ninety-seven cases treated, with practically 100 per cent. of cures.—*Cosmos.*

